# The NLRP1 inflammasome in skin diseases

**DOI:** 10.3389/fimmu.2023.1111611

**Published:** 2023-02-23

**Authors:** Marc Burian, Morna F. Schmidt, Amir S. Yazdi

**Affiliations:** Department of Dermatology and Allergology, RWTH University Hospital Aachen, Aachen, Germany

**Keywords:** inflammasomes, nucleotide-binding domain and leucine-rich repeat containing gene family, interleukin-1β, caspase-1, keratinocytes, skin, innate immunity, therapeutic target

## Abstract

Healthy human skin is constantly exposed to sterile and microbial agents. The skin immune system plays an important role in immune surveillance between tolerance and immune activation. This is mainly mediated by neutrophils, macrophages and most importantly lymphocytes. Keratinocytes, which form the outer skin barrier (epidermis) are also critical for cutaneous homeostasis. Being a non-professional immune cell, recognition of danger signals in keratinocytes is mediated by innate immune receptors (pattern recognition receptors, PRR). While Toll-like receptors are located on the cell membrane or the endosomes, nucleotide-binding domain and leucine-rich repeat containing gene family receptors (NLR) are intracellular PRRs. Some of these, once activated, trigger the formation of inflammasomes. Inflammasomes are multiprotein complexes and serve as platforms that mediate the release of innate cytokines after successful recognition, thereby attracting immune cells. Moreover, they mediate the pro-inflammatory cell death pyroptosis. Best characterized is the NLRP3 inflammasome. The function of inflammasomes differs significantly between different cell types (keratinocytes versus immune cells) and between different species (human versus mouse). In recent years, great progress has been made in deciphering the activation mechanisms. Dysregulation of inflammasomes can lead to diseases with varying degrees of severity. Here we focus on the structure, function, and associated pathologies of the NLRP1 inflammasome, which is the most relevant inflammasome in keratinocytes.

## From the scratch

Keratinocytes not only form the outer structural barrier of human skin but also express molecules that are actively involved in immune responses, such as the secretion of proinflammatory cytokines (1). The recognition of molecules frequently found in pathogens (pathogen-associated molecular patterns [PAMPs]) or non-microbial danger-associated molecular patterns (DAMPs) is sensed by pattern recognition receptors (PRRs) on the surface or intracellularly (2–4). While Toll-like receptors are located on the cell surface or the endosome, intracellular recognition of danger signals triggers the activation of nucleotide-binding domain and leucine-rich repeat containing gene family receptors (NLR) (formerly referred to NOD-like receptors) (5, 6). Some extracellular stimuli such as ATP or Flagellin can activate NLRs *via* specific receptors, while NLRs, such as NLRP1 and NLRP3 can form intracellular protein complexes, so-called inflammasomes. Inflammasomes activate caspase-1, which cleaves cytokines of the innate immune system (mainly IL-1β and IL-18). Once being unconventionally secreted, these cytokines induce a cascade which attracts immune cells (7, 8). Additionally, inflammasome activation can initiate a lytic form of cell death called pyroptosis (9, 10).

All known inflammasomes usually consist of an intracellular sensor (NLRP1, NLRP3, NLRC4, AIM2, Pyrin or CARD8) and can recruit a proinflammatory caspase (cysteine protease). Some require an additional adaptor protein to be able to bind caspases *via* CARD-CARD interactions, and can therefore be viewed as a cytosolic hub that recognizes a signal and subsequently triggers an inflammatory response (8, 11–16).

After activation by stress or danger signals, oligomerization with the adaptor molecule ASC (apoptosis-associated speck-like protein containing a CARD) occurs. As the ASC specks can be visualized in the cytosol, this is used as a read-out for inflammasome activation (17–19). ASC consists of one caspase recruitment domain (CARD) and one pyrin domain (PYD). Through its CARD domain, ASC interacts with pro-caspase-1, which in turn leads to dimerization of the caspase with subsequent self-activation (12, 20, 21). In humans, 12 inflammatory caspases have been identified, distinguishing initiator and effector caspases (the latter involved in apoptosis) (22). Caspase-1, the most well characterized inflammatory caspase, is expressed as an inactive pro-caspase-1 consisting of a CARD domain and subunits p20 and p10 (catalytically active domains). Dimerization and autoproteolytic processes induce its activation and the cleavage of the N-terminal CARD domain (23–26). Substrates of active caspase-1 are pro-IL-1β and pro-IL-18, which are cleaved into their biologically active forms (27). Furthermore, caspase-1 processes Gasdermin D (GSDMD), which is incorporated into the cell membrane leading to pyroptosis *via* pore formation. Secreted and activated proinflammatory cytokines can enter the extracellular component, bind to their receptors, and attract immune cells. The loss of membrane integrity, possibly due to the formation of GSDMD pores in organellar membranes, contributes to pyroptotic cell death (28, 29).

In the past years, inflammasomes have been studied mainly in immune cells (macrophages or dendritic cells) where NLRP3 (nucleotide-binding domain and leucine-rich repeat pyrin domain containing protein 3) was the focus of interest due to its involvement in neurodegenerative or cardiovascular diseases and cancer (30–32). Additionally, other NLRP3-associated hereditary autoinflammatory syndromes from the group of cryopyrin-associated periodic syndromes including Muckle-Wells-Syndrome, familial cold autoinflammatory syndrome and chronic infantile neurological cutaneous articular syndrome have been classified (33). However, inflammasomes are also expressed in epithelial cells such as keratinocytes. Here, NLRP1 (nucleotide-binding domain and leucine-rich repeat pyrin domain containing protein 1) appears to play a central role (34–36). Innate cytokines (IL-1α, IL-1β and IL-18) are constitutively expressed in keratinocytes, unlike in myeloid cells. For this reason, keratinocytes do not need a priming step by PAMPs (LPS) or cytokines (TNF-α, IFN-γ or IL-1) to induce pro-IL1 cytokines or the NLRs (37–40). In this review, we present the basic features of the structure and function of the NLRP1 inflammasome to understand the pathogenesis of cutaneous diseases in particular.

## From form to function

**Figure 1:**
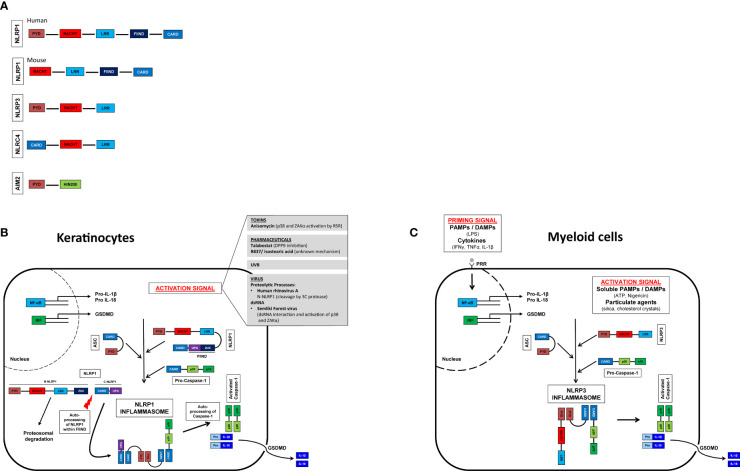
Inflammasome activation in myeloid cells and keratinocytes and their domain structure. Domain structure of human and murine NLRP1 (nucleotide-binding domain and leucine-rich repeat [NLR], pyrin domain [PYD] containing protein 1), NLRP3 (NLR family PYD domain containing protein 3), NLRC4 (NLR family caspase CARD [Caspase recruitment domain] domain containing protein 4), AIM2 (Absent in melanoma 2). GSDMD, gasdermin D **(A)**. Activation signals lead to oligomerization of the sensor (NLRP1/NLRP3) with ASC and caspase 1. Self-activation of caspase 1 and subsequent activation of pro-IL-1β and pro-IL-18. Pore formation in the cell membrane allows secretion of mature IL-1β and IL-18. Myeloid cells **(C)**, unlike keratinocytes **(B)**, do not constitutively express pro-IL-1β and pro-IL-18 and therefore require a priming step (41, 42).

Human NLRP1 (hNLRP1) consists of an N-terminal pyrin domain (PYD) followed by a NACHT domain and leucine-rich repeats (LRRs). At the C-terminal end of hNLRP1 there is a function-to-find domain (FIIND) and a CARD (**Figure 1A**) that are autoproteolyzed between the ZU5 and UPA subdomains (43, 44). Constitutive autoproteolysis between the two subdomains is required for NLRP1 activation, but is not sufficient on its own (45, 46). Despite constitutive autoproteolysis, the amino-terminal fragment of NLRP1 (N-NLRP1) remains associated with the C-terminal effector fragment (C-NLRP1), promoted by interaction with dipeptidyl peptidase (DPP) 9 (47, 48). Activation signals such as UVB (38) induce the proteosomal degradation of N-NLRP1, which thus can no longer inhibit the C-NLRP1. The CARD-domain of C-NLRP1 recruits ASC and caspase-1 to form the NLRP1 inflammasome (49).

In contrast to humans, mice express two NLRP1 paralogs (NLRP1a and NLRP1b) and one pseudogene (NLRP1c) all of which lack the N-terminal PYD domain (**Figure 1A**) (50, 51). Interestingly, UVB irradiation of murine keratinocytes does not lead to inflammasome activation; expression of pro-IL-1ß and NLRP1 is only marginal (52). Unlike human keratinocytes, which secrete IL-1ß upon UVB irradiation, this appears to be mediated by a yet unidentified cell type. Therefore, murine models investigating NLRP1-activation might not be transferable to the human system. Murine NLRP1b is activated by bacterial enzymes, as for example the E3 ubiquitin ligase IpaH7.8 of *Shigella flexneri* or the protease lethal factor of *Bacillus anthracis* (53, 54).

In contrast to murine keratinocytes and human myeloid cells, human keratinocytes do not require an initial “priming” signal but can be activated directly by one stimulus. Among the best studied activation mechanisms are (i.) proteolytic processes, (ii.) DPP9 inhibition, (iii.) double-stranded RNA, and (iv.) phosphorylation (35). Briefly, the human rhinovirus (HRV) 3C protease cleaves hNLRP1 between the PYD and NACHT domains and subsequently the newly formed amino terminus of NLRP1 is ubiquitinated and degraded by the proteasome (55). NLRP1 is also activated by the anticancer drug talabostat (Val-boroPro, PT-100) (56). At steady state DPP9 binds to one fragment of N-NLRP1 and two fragments of C-NLRP1. Talabostat releases C-NLRP1 from DPP9, leading to proteasomal degradation of N-NLRP1 (47, 48, 57, 58). Double-stranded RNA (dsRNA) from alphaviruses can bind to the LRR domain, most likely leading to a conformational change of N-NLRP1 and proteasomal degradation and consequently activation of C-NLRP1 (58). UVB and certain bacterial toxins activate the ribotoxic stress response (RSR) and in turn induce NLRP1 phosphorylation between PYD and NACHT domains *via* ZAKα (a MAPK kinase activated by the RSR) and downstream p38. Phosphorylation results in ubiquitination and proteasomal degradation of N-NLRP1 (55, 59).

In summary, similarly to the NLPR3 inflammasome in myeloid cells, the NLRP1 inflammasome in keratinocytes is activated by a variety of microbial or sterile danger signals, leading to de-stabilization of the N-terminal fragment of NLRP1 and, in turn, driving NLRP1 activation (**Figure 1B**). As inflammasome activation leads to the secretion of highly potent pro-inflammatory innate cytokines, all inflammasomes are strictly regulated. Dysregulation of inflammasomes of their downstream receptors or their antagonists sometimes leads to severe diseases. While a putative involvement of NLRP3 in numerous pathologies have been described for NLRP3 in recent years (**Figure 1C**), the exact role of NLRP1 or inflammasomes in the human skin immune system *in vivo* is poorly understood and needs further investigation.

## From health to disease

The intrinsic inborn function of inflammasomes is to maintain the balance of the organism in the defense against pathogens or non-microbial danger signals (60). Impairment of inflammasome regulation is known in many common and less common diseases in the human system. There are diseases which are associated with certain inflammasome subtypes. NLRP3 inflammasome for example is associated with frequent diseases such as artherosclerosis and Alzheimer’s disease, most probably activated during the frustrated phagocytosis of cholesterin or fibrillar β-amyloid proteins (31, 61). Once activated, the secretion of IL-1β perpetuates inflammation.

When looking at inflammasome-associated diseases, it becomes obvious that there are above mentioned tissue-specific differences in function, as organ-specific symptoms can occur without a systemic impairment (62). As stated above, NLRP1 is the most prominent inflammasome sensor in the skin (63). Its function is not strictly linked to the innate immune system, as common inflammatory skin diseases (e.g. psoriasis and atopic dermatitis) are associated with the NLRP1 inflammasome and more importantly proinflammatory cytokines from IL-1 family (36, 60, 64–66). Atopic dermatitis is a prototypical disease that is shaped by genetic and environmental influences. A single nucleotide polymorphism (SNP) in the NLRP1 gene from Swedish patients with atopic dermatitis was classified as a possible susceptibility for the occurrence of a disease manifestation, although not the only contributing factor (67). Similarly, SNPs in the NLRP1 gene have been studied in patients with psoriasis vulgaris and have shown a strong association with disease susceptibility (68). In addition, the impaired endogenous production of IL-1 by keratinocytes also seems to play a role in the pathogenesis of psoriasis. Here, the dysbalance of the skin microbiome could be identified as a main cause (36, 69). A closer look at autoimmune diseases reveals the double-edged role of inflammasomes. On the one hand, activation is important for pathogen and sterile defense, especially at border surfaces, such as the epithelia of the skin and the gastrointestinal tract; on the other hand, overactivation and dysbalance can lead to disease manifestation in almost any organ [reviewed in (60)]. With regard to the skin, a classic inflammasome-associated disease is vitiligo, a multifactorial disease that is based on abnormal melanocyte function. Several SNPs in the NLRP1 gene were reported to be associated with vitiligo (70) and patient-derived monocytes were shown to secrete increased amounts of IL-1β upon activation (71). A putative role of IL-1 cytokines is also described in systemic lupus erythematosus (72, 73), leprosy (74, 75), oral pemphigus vulgaris (76) and Addison’s disease (hyperpigmentation) (77, 78). However, some of these studies lack the clear demonstration of the functional pathogenetic role of NLRP1, as they only describe genetic findings in the way of SNPs.

A functional connection between diseases and inflammasomes was shown in autoinflammatory diseases, where the term inflammasomopathies was shaped. It is an emerging group of multisystemic diseases characterized by elevated levels of proinflammatory cytokines in the serum, some of which present with cutaneous symptoms (79–82). Several of those autoinflammatory diseases with skin manifestations have been described in which germline-activating mutations in the inflammasome sensor NLRP1 are present. Depending on the site in which the mutations occur, clinical pictures vary from localized cutaneous symptoms to accompanying systemic reactions with fever (63). In multiple self-healing palmoplantar carcinoma (MSPC), gain-of-functions are present in the pyrin domain of NLRP1 (63). Clinically, ulcerated, hyperkeratotic nodules termed keratoacanthomas are seen predominantly on the palmoplantar skin as well as on the conjunctiva and corneal epithelium. These lesions normally regress spontaneously but can predispose to the development of squamous cell carcinoma (SCC), demonstrating a link between inflammasome and carcinogenesis. Another disease with gain-of function mutations in the LRR domain of NLRP1 is familial keratosis lichenoides chronica (FKLC), in which affected individuals show disseminated lichenoid papules predominantly on the extremities and trunk. In both cases, the mutations lead to a loss of the autoinhibitory effect in NLRP1 with a consecutive increase in proinflammatory cytokines (63). Secretion of IL-1 cytokines implies the release of further proinflammatory cytokines and growth factors creating a proinflammatory milieu. This manifests in focal epidermal hyperplasia and hyperkeratosis, one of the morphological hallmarks of psoriasis (63, 68).

A further disease called NLRP1-associated autoinflammation with arthritis and dyskeratosis (NAIAD) is coined by a mutation between the NACHT and the LRR region or in the FIIND domain. It is accompanied by systemic reactions, especially fever, and high levels of proinflammatory cytokines in the blood (83).

A promising therapeutic approach in treatment of systemic autoinflammatory syndromes used in clinical practice is the blockade of the IL-1 pathway. Here, three modalities are available: the IL-1 receptor antagonist anakinra, the fusion protein rilonacept or canakinumab, a human monoclonal antibody directed against IL-1β (84–86).

The last two decades elucidated several diseases associated with mainly NLRP3 inflammasome dysregulation or IL-1 production and it remains to be found out why certain inflammasome subtypes lead to a distinct clinical picture. Although NLRP1 is also highly expressed in myeloid cells, the germline mutations have a more severe phenotype in the skin compared to systemic functions (63). It is still currently under investigation if this might be due to unknown, non-specific mutations, e.g. in non-coding areas or through post-translational modifications.

There is also growing evidence of overlapping autoimmune- autoinflammatory mechanisms. Innate and adaptive immune processes cannot be strictly separated from each other, precisely because they show similarities in the pathophysiology of clinically different diseases (87).

Gain-of function mutations in NLRP1 inflammasome demonstrate the link between inflammasome dysfunction and carcinogenesis (63). Inflammation and recognition of tumor cells by the immune system is crucial to control tumor growth. In clinical practice, inducing inflammation is used to treat superficial non-melanoma skin cancer (88). However, proinflammatory processes also drive tumorigenesis, for instance in lichen planus or chronic wounds (89). UVB radiation is the major risk factor for nonmelanoma skin cancer (90) as well as a known activator of the NLRP1 inflammasome in human keratinocytes leading to secretion of proinflammatory cytokines (38). Additionally, it results in decreased levels of anti-apoptotic proteins (Bcl-2 and Bcl-XL) (91). Other recent reports present downregulation of inflammasome components in SCC, which may contribute to tumor progression, especially at later time points (92). One might postulate different functions of innate immunity or inflammasomes at different stages of disease progression. In tumorigenesis, inflammation might favor tumor progression, while once the tumor is established, innate immunity might control tumor growth or even induces tumor regression. The proinflammatory anti-carcinogenic effect of inflammasomes is also exploited in the topical therapy of early SCC or its precursor, actinic keratosis. Imiquimod which is used as a topical treatment is both an activator of TLR7 and of NLRP3 (93, 94). In primary malignant melanoma, a pro-tumorigenic role of the NLRP3 inflammasome was shown (95), while anti-apoptotic influence of the NLRP1 inflammasome seems to play a role particularly in metastatic malignant melanoma (96).

Besides the previously discussed cancers, rare mutations associated with DPP9 deficiency should also be mentioned. Here, not only immunological and neurological deficits, but skin changes can also occur in the setting of DPP9 deficiency (97). In addition, a mutation leading to impaired binding of DPP9 to the inflammasome, resulting in loss of autoinhibition with enhanced activation was recently described (16). The clinical relevance and the question of why NLRP1 deficiencies or functional disorders lead to isolated skin manifestations is still the subject of current research.

**Figure 2:**
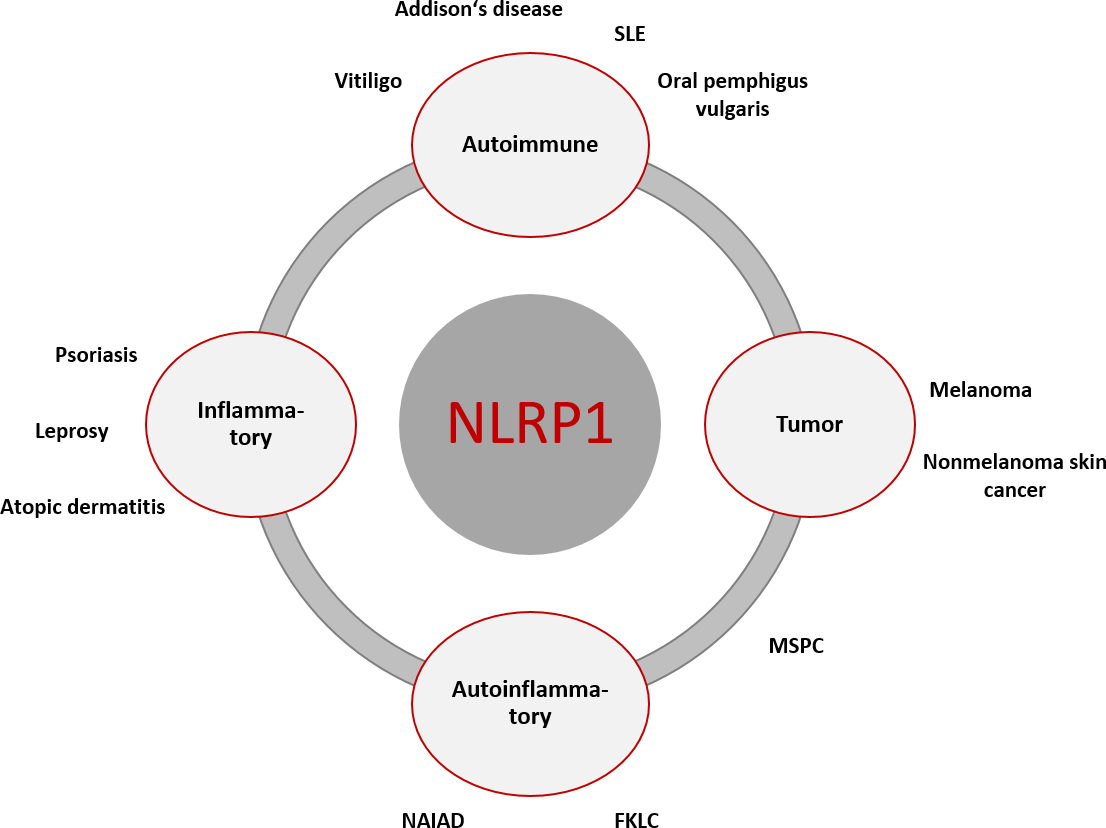
Overview of NLRP1-associated diseases with primary cutaneous manifestation or associated cutaneous clinical symptoms. The primary genesis may be inflammatory, autoimmune, autoinflammatory, and cancerogenic. There is overlap between the different entities, which can lead to symptom complexes that are not clearly assignable diagnostically and therapeutically. [FKLC, familial keratosis lichenoides chronica; MSPC, Multiple Self-Healing Palmoplantar Carcinoma; NAIAD, NLRP1- associated autoinflammation with arthritis and dyskeratosis; SLE, systemic lupus erythematodes].

For an overview of the diseases mentioned, see **Figure 2**.

## From now to future

Since the discovery of inflammasomes in 2002 (8) there have been major advances in understanding how PAMPs and DAMPs are recognized by the immune system and how it triggers both innate and adaptive inflammation. Likewise, during the past two decades, the impact of IL-1, inflammatory caspases or inflammasomes on various human diseases, such as gout, arteriosclerosis, pyoderma gangrenosum became more apparent, with a still growing list of diseases (62). The investigation of the activation and regulation of inflammasomes at molecular levels is most advanced in monogenic hereditary syndromes described above, where a targeted treatment is now possible (98). The effect of anti-IL-1 treatments in other non-monogenetic disorders is still under investigation (99). However, some essential questions have not yet been answered. With regard to the skin, no physiologically relevant activator of NLRP1 inflammasomes has been identified so far, apart from UVB (38). In the future, it will be important not to overlook the role of commensal microorganism on the skin and their secreted products (such as metabolites but also secreted colonization/virulence factors). A challenge will be to differentiate the effect of commensals or pathogens on the NLRP1 inflammasome in the skin. Microbe-specific virulence factors could have distinct effects on the fine regulation of inflammasome activity. It is yet cryptic if NLRP1 activation in keratinocytes itself, the subsequent activation of inflammatory caspases or the secretion of innate cytokines impair the skin barrier or induce the recruitment of inflammatory cells to the epidermis. As the skin is involved in most of the “inflammasopathies” and inflammasome activation was reported in various diseases, NLRP1 inflammasome might be a promising pathway to develop targeted therapies. Future inflammasome studies should primarily be investigated in the human model to depict physiological conditions.

## Author contributions

MB, MS and AY contributed to the manuscript writing. All authors contributed to the article and approved the submitted version. 
